# Sleep, Physical Activity, and Diet of Adults during the Second Lockdown of the COVID-19 Pandemic in Greece

**DOI:** 10.3390/ijerph18147292

**Published:** 2021-07-08

**Authors:** Zisis Papazisis, Pantelis T. Nikolaidis, Georgia Trakada

**Affiliations:** 1 Faculty of Mathematics, School of Sciences, University of Ioannina, 451 10 Ioannina, Greece; zisispap3d@gmail.com; 2 School of Health and Caring Sciences, University of West Attica, 122 43 Athens, Greece; 3 Division of Pulmonology, Department of Clinical Therapeutics, School of Medicine, National and Kapodistrian University of Athens, Alexandra Hospital, 115 28 Athens, Greece; gtrakada@hotmail.com

**Keywords:** exercise, quality of sleep, sex difference, walking, weight status

## Abstract

The aim of the present study was to evaluate the possible correlations between sleep, physical activity, and diet in the general population of Greece during the second lockdown due to COVID-19 pandemic. A web-based questionnaire including 28 items was completed by 494 adults (age 31.5 ± 12.4 years). Half of the participants (49.8%) reported good, 44.1% moderate, and 6.1% bad quality of sleep, whereas 17.2% answered that the quality of sleep improved, 56.5% did not change, and 26.3% worsened compared to a normal week. Compared to normal, sleep duration in lockdown increased by 0.24 h (95% CI; 0.13, 0.35; *p* < 0.001, d = 0.198). More under-weight (32.4%) and obese (34.1%) respondents reported worsened quality of sleep in lockdown compared with normal (25.6%) and overweight participants (22.7%) (*p* = 0.006, Cramer’s φ = 0.191). A small effect for BMI group on sleep duration was observed (*p* = 0.011, η^2^ = 0.023), where overweight and obese slept less (–0.44 h and –0.66 h, respectively) than normal weight participants. Subjects with the highest percentage of increased food consumption reported decreased sleep duration (*p* = 0.012) and worsened sleep quality (*p* = 0.003). Compared with a normal week, physical activity of a high and moderate intensity decreased for 43.0% and 37.0% of participants, did not change in 32.9% and 36.1% of participants, and increased in 24.1% 26.9%, respectively, whereas walking time decreased in 31.3%, did not change in 27.3%, and increased in 41.5% of participants. Increased high and moderate intensity physical activity was related with an improvement in sleep quality (*p* < 0.001). Those with decreasing walking time reported the highest percentage of decreased sleep quality (*p* = 0.006) and worsened sleep quality (*p* = 0.016). In conclusion, both quality and quantity of sleep were impaired during the second lockdown and the observed changes were associated with diet and physical activity.

## 1. Introduction

The COVID-19 pandemic and the accompanying lockdown have impacted many aspects of the daily life of adults, including sleep duration and quality [1,2,3]. For instance, a study on U.S. Americans showed that ~37% had increased sleep duration, whereas ~17% had decreased duration [2], and 45% reported a worsening of sleep quality in research on Italians [4]. Changes in sleep duration were also associated with occupation, as suggested by research reporting no change in health professionals and an increase in the other professionals [3]. In addition, it was observed that hospitalized COVID-10 patients had obesity as the second most common condition [5,6], highlighting the role of weight status. In addition, sleep duration might regulate body weight through its impact on two key hormones (ghrelin and leptin) for appetite regulation [7]. Nevertheless, little information existed on the association of sleep characteristics with weight status, physical activity (PA), and food consumption during lockdown.

According to the International Classification of Sleep Disorders, insomnia disorders, sleep-related breathing disorders, central disorders of hypersomnolence, circadian rhythm sleep-wake disorders, sleep-related movement disorders, parasomnias, and other sleep disorders are categories of sleep disorders [8]. Factors related to sleep quality include sex (men), marital status (married), PA, education, healthy social relations, socioeconomic class, income (positive role), age, pet owners, caffeine intake, smoking, stress, and irregular sleep-wake patterns (negative role) [9,10].

It has been observed that people with obesity presented worsened sleep in lockdown [11]. A shorter sleep duration is a predictor of weight gain, as suggested in a systematic scoping review [12]. Moreover, those with negative changes in sleep quality reported more weight gain than those with positive changes [4]. With regards to the relationship of sleep duration with weigh status in Australians, it was found that the odds of overweight/obesity were the greatest for those who slept <6 h, and the risk of overweight/obesity decreased for those with sleep >7 h [13]. Moreover, compared with recommended sleep time (i.e., ≥7 to ≤9 h) in U.S., American black women who were very short sleepers (≤6 h) and long sleepers (>9 h) had a significantly greater body mass index (BMI) [14]. A longer sleep duration was related with a lower BMI in Chinese freshmen [15]. In university students, being overweight showed a higher odds ratio of less sleep than normal weight students [16]. In addition, the relationship between sleep duration and BMI depended on age [17,18], and consequently, age should be considered in studies of sleep characteristics and weight status.

Lack of PA and/or increased food consumption are the most prominent risk factors for an increase in BMI [19]. PA is widely considered to improve sleep quality and is often proposed as a non-pharmacologic treatment to improve sleep [20]. In a recent review [21], it was demonstrated that both acute and regular PA improved sleep quality. The positive effects were generally preserved across lifespan, independently of sex, in a dose–response relationship regarding length, but not intensity, of exercise. PA improved sleep even in patients suffering from insomnia or sleep apnea. Diet is also widely considered an important modifiable factor that has been often proposed to improve sleep duration and quality [22]. Eating schedules, food intake, and energy balance altered the propensity for sleep and sleep architecture [23]. Adults consuming proinflammatory diets are more likely to have a short or long sleep duration, and/or self-reported sleep disturbances [24]. Moreover, a high-fat food intake enhances sleepiness and deteriorated sleep apnea [25].

No information exists so far about these implications during COVID-19 pandemic. Such information would be important for healthcare professionals working with patients with sleep disorders. Therefore, the aim of the present study was to evaluate the possible correlations between sleep and BMI in relation to PA and food intake during the second restrictive measures due to the COVID-19 pandemic in Greece.

## 2. Materials and Methods

### 2.1. Study Design

The study design is cross-sectional, where data were collected from 22 March to 7 April 2021 using a questionnaire. This period was characterized by restrictive measures in all domains of daily life, including e-working, e-education, and limited transportation [26]. Because of the lockdown measures during the period of the study, the administration of a paper-and-pencil questionnaire was not feasible. A web-based (Google Forms [27]) questionnaire including 28 items was promoted through social media [28] and was completed by a convenience sample of 500 participants. Inclusion criteria were sex and adult age (≥18 years). Six participants were excluded because of their age (younger than 18 years)m resulting in a final sample of 494 adults (age 31.5 ± 12.4 years) who were further analyzed.

### 2.2. Ethics Approval

The study was approved by the local institutional review board (Alexandra University Hospital, Athens, Greece; approval number 232/3 April 2020). The participants were recruited using social media and—prior to answering the questionnaire—all participants provided informed consent after being informed about the aims and details of the study. It was highlighted in the call for participation in the present study that participation was voluntarily and participants could withdraw at any moment.

### 2.3. Questionnaire

The questionnaire was available in Greek and its contents are presented in Supplementary Materials. The items about the sociodemographic and sleep characteristics were used previously in a recent study [3]. The items on PA were based on the short version of the International Physical Activity Questionnaire, which has been tested for validity and reliability [29,30]. In addition, a good agreement was observed between self-reported and direct anthropometric measurements, suggesting the further use of self-reported height and weight data for research [31,32].

### 2.4. Statistical Analysis

All of the statistical and data analyses were performed using IBM SPSS v.26.0 (IBM SPSS Statistics for Windows; Armonk, NY, USA). The figures were created by GraphPad Prism v.7.0 (GraphPad Software, San Diego, CA, USA). Descriptive statistics (mean, standard deviations, and frequencies) were calculated for all of the variables. Participants were classified into two age groups, <43 (total, *n* = 382; women, *n* = 225; men, *n* = 157) and >43 years (total, *n* = 112; women, *n* = 71; men, *n* = 41). In addition, they were grouped into four BMI categories according to the classification of the World Health Organization [33], as follows: under-weight (BMI under 18.5 kg·m^−2^; total, *n* = 34; women = 34), normal-weight (BMI from 18.5 to 24.9 kg·m^−2^; total, *n* = 289; women, *n* = 200; men = 89), overweight (BMI from 25.0 to 29.9 kg·m^−2^; total, *n* = 128; women, *n* = 48; men = 80), and obese (BMI greater than 29.9 kg·m^−2^; total, *n* = 41; women, *n* = 14; men = 27). A two-way analysis of variance examined the main effects of sex and age group (<43 vs. >43 years) and their interaction on sleep duration. Eta square (η^2^) examined the magnitude of differences in ANOVAs. A dependent *t*-test examined the differences in sleep duration between lockdown and normal. Cohen’s d evaluated the magnitude of difference in t-test. A between-within subject ANOVA examined the effect of time (normal versus lockdown) and the sex × lockdown interaction—as well as the age group × lockdown interaction within each sex—on sleep duration. A one-way ANOVA evaluated the differences in sleep duration among BMI groups, and a two-way ANOVA tested the sex × BMI group interaction on sleep duration. Chi-square (χ^2^) evaluated associations among non-parametric data and Cramér’s phi (φ) assessed the magnitude of such associations. Statistical significance was set at alpha = 0.05.

## 3. Results

### 3.1. Demographic Data, Sleep, and Physical Activity

**Figure 1:**
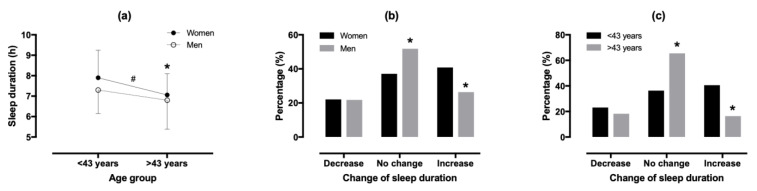
Sleep duration by sex and age group (**a**), change of sleep duration in lockdown compared with normal according to sex (**b**) and age group (**c**). (**a**) * difference between age groups at *p* < 0.05, # sex difference at *p* < 0.05; (**b**) * sex × change of sleep duration association at *p* < 0.05; (**c**) age group × change of sleep duration association at *p* < 0.05.

The demographic data, sleep, and PA can be seen in Table 1. In lockdown, women slept more than men by 0.50 h (7.70 ± 1.33h versus 7.20 ± 1.22 h; 95% confidence intervals (CIs), 0.27, 0.73; *p* < 0.001, Cohen’s d = 0.392). Younger participants slept more than older participants by 0.69 h (7.65 ± 1.30 h versus 6.96 ± 1.19 h; 95% CIs, 0.42, 0.96; *p* < 0.001, Cohen’s d = 0.554). No sex × age group interaction on sleep duration was found (*p* = 0.221, η^2^ = 0.003; Figure 1).

Compared with a normal week, sleep duration in lockdown decreased for 22.0%, did not change for 42.9%, and increased for 35.1% of participants. A sex × lockdown association was observed with more women increasing sleep duration than men (χ^2^ = 12.777, *p* = 0.002, φ = 0.162). An age × lockdown association was shown with younger participants having an increased sleep duration compared with their older counterparts (χ^2^ = 31.975, *p* < 0.001, φ = 0.256).

**Figure 2:**
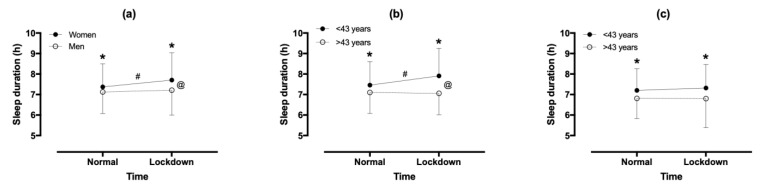
Sleep duration in normal and lockdown according to sex (**a**) and age group in women (**b**) and men (**c**). (**a**) * sex difference at *p* < 0.05, # difference between normal and lockdown at *p* < 0.05, @sex × time (normal versus lockdown) interaction at *p* < 0.05; (**b**,**c**) * difference between age groups at *p* < 0.05, # difference between normal and lockdown at *p* < 0.05, @age group × time (normal versus lockdown) interaction.

Compared with normal, sleep duration in lockdown increased by 0.24 h (95% CI; 0.13, 0.35; *p* < 0.001, d = 0.198) (Figure 2). A sex × lockdown interaction on sleep duration was observed (*p* = 0.035, η^2^ = 0.009), where sleep duration increased in women, but not in men. In women, an age group × lockdown interaction on sleep duration was shown (*p* = 0.007, η^2^ = 0.025), where sleep duration increased for participants aged <43 years, but not >43 years. In men, no age group × lockdown interaction was found (*p* = 0.590, η^2^ = 0.002).

Half of participants (49.8%) reported good, 44.1% moderate, and 6.1% bad quality of sleep, whereas 17.2% answered that the quality of sleep improved, 56.5% did not change, and 26.3% worsened compared with a normal week. Neither a sex × quality of sleep association (χ^2^ = 1.225, *p* = 0.542, φ = 0.050) or an age group × quality of sleep association (χ^2^ = 0.016, *p* = 0.992, φ = 0.006) were observed. A sex × change of quality of sleep association (χ^2^ = 6.550, *p* = 0.038, φ = 0.115) was shown, with more women reporting a change (either worsening or improvement) in quality of sleep than men. No age group × change of quality of sleep association (χ^2^ = 4.601, *p* = 0.100, φ = 0.097) was found.

### 3.2. Sleep Characteristics by Body Mass Index Group

**Figure 3:**
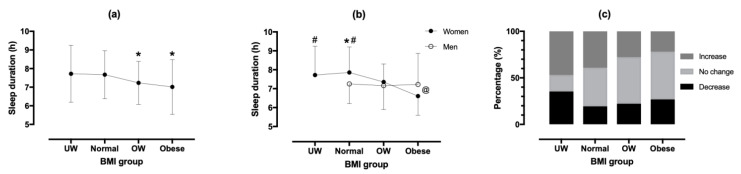
Sleep duration by body mass index group (**a**) and sex (**b**), and change of sleep duration (**c**). (**a**) * difference from normal-weight group at *p* < 0.05; # difference from Obese group at *p* < 0.05, * sex difference at *p* < 0.05, @sex × BMI group interaction at *p* < 0.05. UW—under-weight; OW—over-weight.

A small main effect of BMI group on sleep duration was observed (*p* = 0.011, η^2^ = 0.023), where overweight and obese participants slept less (−0.44 h and −0.66 h, respectively) than normal weight participants (Figure 3). A BMI group × sex interaction on sleep duration in lockdown was shown (*p* = 0.017, η^2^ = 0.017), where sleep duration differed among BMI groups in women (*p* = 0.001, η^2^ = 0.053), but not in men (*p* = 0.881, η^2^ = 0.001). A BMI group × change of sleep duration association was observed (χ^2^ = 17.987, *p* = 0.006, φ = 0.193), with more under-weight, overweight, and obese participants experiencing decreased sleep duration than their normal-weight peers. More under-weight (32.4%) and obese (34.1%) participants reported a worsened quality of sleep in lockdown than normal (25.6%) and overweight participants (22.7%) (*p* = 0.006, Cramer’s φ = 0.191).

### 3.3. Change in Weight, Physical Activity, and Food Consumption

Compared with a normal week, PA of high and moderate intensity decreased for 43.0% and 37.0%, did not change for 32.9% and 36.1%, and increased for 24.1% 26.9% of participants, respectively, whereas the time walking decreased for 31.3%, did not change for 27.3%, and increased for 41.5% of participants.

Δweight was associated with a change in PA of high (χ^2^ = 54.980, *p* < 0.001) and moderate intensity (χ^2^ = 47.099, *p* < 0.001), and walking (χ^2^ = 22.342, *p* < 0.001), where those with a decreased weight reported a larger increase of PA of high and moderate intensity, and walking compared to those with no change or gain of weight (Table 2). The magnitude of these associations was larger in PA of high and moderate PA than in walking. In addition Δweight was associated with a change in nutrition quantity (χ^2^ = 265.264, *p* < 0.001) and quality (χ^2^ = 124,148, *p* < 0.001), where those with decreased weight reported decreased food consumption and improved quality of nutrition than those with no change or gain of weight. The magnitude of these associations was larger in the quantity than in the quality of nutrition.

### 3.4. Change of Sleep Characteristics, Weight, Physical Activity, and Food Consumption

Δweight was not related with sleep duration (*p* = 0.316), whereas the association of Δweight with sleep quality was close to statistical significance (*p* = 0.068), with those with weight gain reporting the highest percentage of sleep quality worsening (Table 3). With regards to nutrition, change in food quantity was associated with sleep duration (*p* = 0.012) and sleep quality (*p* = 0.003), where those with the highest percentage of increased food consumption reporting decreased sleep duration and worsened sleep quality. Similarly, those with the highest percentage of worsened food quality reported decreased sleep duration and worsened sleep quality. Finally, increasing high and moderate intensity PA was related with improvement in sleep quality (*p* < 0.001), but not with changes in sleep duration (*p* = 0.628 and *p* = 0.376, respectively). Those with decreasing walking time reported the highest percentage of decreased sleep quality (*p* = 0.006) and worsened sleep quality (*p* = 0.016).

## 4. Discussion

The present study depicted that lockdown due to the COVID 19 pandemic affected sleep, physical activity, and food consumption in the general population of Greece. Sleep quality deteriorated when compared with a normal week. A worse quality of sleep was reported more often by under-weight and obese participants than normal and overweight participants. Furthermore, decreased physical activity and increased food consumption were associated with the aggravation of sleep quality.

During the COVID-19 pandemic, Greek authorities applied restrictive measures like public commuting and travelling restrictions, educational institutes’ closure, and the enforcement of tele-working practices in two periods (first lockdown from 23 March 2020 for 43 days and the second lockdown from 7 November 2020 for 210 days). The measures put in place in Greece were among the strictest in Europe, and people were experiencing sudden and major changes in their daytime routines. All parts of life were affected, including sleep, physical activity, and diet.

The observed increase in sleep duration and the affected quality of sleep during lockdown in our study were in agreement with previous research [34,35]. The impact of lockdown on sleep characteristics was more pronounced in the younger participants in the present study, which was also in line with previous research [2]. An explanation of the impact of the lockdown on sleep characteristics of the younger age group was that this age group might be characterized by more anxiety as a result of less stable socio-economic status compared with older participants [2,36]. Nevertheless, it should be highlighted that the mean sleep duration of participants was within the physiological range of 7–8 h [37]; thus, the focus of the present study was more on the variation of sleep duration rather than the mean score.

Our findings that overweight and obese participants slept less than normal weight participants, and that more under-weight and obese participants reported a worsened quality of sleep in lockdown than normal and overweight participants indicated a favorable profile of normal-weight status for sleep. It is well documented that obesity is associated with decreased sleep duration and poor sleep quality [38]. Although sleep is a completely sedentary activity, it does not lead to weight gain [39]. Both a short and long sleep duration were associated with obesity risk [40]. Moreover, a decrease in sleep duration was associated with an increased energy and fat intake [41]. The association of decreased sleep duration with increased weight might be attributed to biological mechanisms including hormonal changes and metabolites [42,43]. For instance, optimal sleep duration was related to less desire for high calorie foods in overweight young adults [44]. Furthermore, pathological conditions associated with obesity could lead to unrefreshing and short sleep, like obstructive sleep apnea (OSA) [39].

The COVID-19 pandemic had affected weight-related behaviors, including healthy eating and physical activity, especially among adults with high BMI [45]. Moreover, limited access to fresh food due to lockdown led towards a greater consumption of highly processed foods, and those with long shelf lives that are usually high in salt, sugar, and saturated fat that provide only a transient sensation of fullness [46]. During the last decades, obesity has become a major health issue, with a prevalence of 65–70% in UK and US adult populations [47].

More exercise and less food consumption are the gold standards for normal weight. We demonstrated that even in lockdown, with preserved daytime activities, those with decreased weight reported a larger increase of PA of a high and moderate intensity and walking, decreased food consumption, and improved quality of nutrition compared with those with no change or a gain in weight. Moreover, increased PA and decreased food consumption were associated with an increased quantity and quality of sleep. Previous data also suggest that PA and diet improve sleep, and are often proposed as modifiable, non-pharmacologic treatments for sleep complains [21,22].

A limitation of the present study is that information on smoking and health status was not included in the analysis, and it was acknowledged that smoking, pain, stress, depression, and other health conditions were associated with a poor quality of sleep [48,49]. An association of psychological factors such as level of stress, anxiety, and depression with sleep disorders has also been demonstrated during lockdown [50,51]. With regards to marital status, a traditional classification was used (i.e., married versus non-married), whereas it was recognized that other status could also be included, e.g., people living together but un-married or information about the number of children. On the other hand, the strength of the study was the inclusion data on two major correlates of weight status (i.e., PA and food consumption), which were also shown to be associated with changes in sleep characteristics.

## 5. Conclusions

In conclusion, sleep was impaired during the second lockdown of the COVID19 pandemic. However, participants with adequate physical activity, balanced diet, and normal weight retained a good sleep quality. Considering the findings of the present study, healthcare professionals working with patients with sleep disorders and scientists interested in this field should consider the variation of the impact of lockdown on sleep characteristics according to BMI, diet, and PA.

## Figures and Tables

**Table 1:** Demographic data, sleep, and physical activity (PA) of participants (*n* = 494).

Variable	Outcome	*n*	%
Sex	Women	296	59.9
	Men	198	40.1
Age group	<43 years	382	77.3
	>43 years	112	22.7
Marital status	Unmarried	382	77.3
	Married	112	22.7
Residence	Rural	79	16.0
	Urban	415	84.0
Education	Primary	11	2.2
	Secondary	43	8.7
	Tertiary ^(a)^	439	89.0
Job	Health professionals	46	9.3
	With physical presence	153	31.0
	Full-time distance working	87	17.6
	Part-time distance working	49	9.9
	Unemployed	39	7.9
	Student	67	13.6
	Other	53	10.7
Compliance with measures	Yes	442	89.5
	No	52	10.5
Presence of COVID-19	Yes	114	23.1
	No	380	76.9
BMI group	Under-weight	34	6.9
	Normal-weight	289	58.7
	Over-weight	128	26.0
	Obese	41	8.3
Change of sleep	Decrease	107	22.0
	No change	209	42.9
	Increase	171	35.1
Quality of sleep	Bad	30	6.1
	Average	217	44.0
	Good	246	49.9
Change of quality of sleep	Worsen	130	26.3
	No change	279	56.5
	Improve	85	17.2
Change of high intensity PA	Decrease	200	43.0
	No change	153	32.9
	Increase	112	24.1
Change of moderate intensity PA	Decrease	164	37.0
	No change	160	36.1
	Increase	119	26.9
Change of walking	Decrease	150	31.3
	No change	131	27.3
	Increase	199	41.5
Change of eating (quantitatively)	Decrease	82	16.7
	No change	238	48.4
	Increase	172	35.0
Change of eating (qualitatively)	Decrease	141	28.7
	No change	224	45.6
	Increase	126	25.7

^(a)^ Tertiary refers to university studies.

**Table 2:** Association of changes in body weight with changes in physical activity (PA) and nutrition.

Variable		Body Weight			
		Decrease	No change	Increase	Statistic
High intensity PA	Decrease	31.4	33.7	57.0	χ^2^ = 54.980
	No change	20.9	45.1	27.5	*p* < 0.001
	Increase	47.7	21.1	15.5	φ = 0.342
Moderate intensity PA	Decrease	23.8	25.9	52.5	χ^2^ = 47.099
	No change	31.0	44.6	32.8	*p* < 0.001
	Increase	45.2	29.5	14.8	φ = 0.326
Walking	Decrease	24.4	23.5	41.5	χ^2^ = 22.342
	No change	20.0	31.7	24.6	*p* < 0.001
	Increase	55.6	44.8	33.8	φ = 0.214
Food quantity	Decrease	60.9	8.6	2.5	χ^2^ = 265.264
	No change	29.3	73.3	32.7	*p* < 0.001
	Increase	9.8	18.2	64.8	φ = 0.739
Food quality	Worsen	11.0	17.1	50.3	χ^2^ = 124.148
	No change	28.6	58.3	39.2	*p* < 0.001
	Improve	60.4	24.6	10.6	φ = 0.495

**Table 3:** Association of changes in sleep duration and quality with changes in physical activity (PA) and nutrition.

Variable		Sleep Duration				Sleep Quality			
		Decrease	No Change	Increase	Statistic	Worsen	No Change	Improve	Statistic
Body weight	Decrease	15.4	44.0	40.7	χ^2^ = 4.728	26.1	51.1	22.8	χ^2^ = 8.753
	No change	24.7	44.6	30.6	*p* = 0.316	22.2	63.0	14.8	*p* = 0.068
	Increase	23.9	41.1	35.0	φ = 0.100	32.2	52.8	15.1	φ = 0.135
High intensity PA	Decrease	24.5	41.5	34.0	χ^2^ = 2.593	36.3	53.9	9.8	χ^2^ = 32.096
	No change	20.5	45.5	34.0	*p* = 0.628	18.4	63.3	18.4	*p* < 0.001
	Increase	19.3	40.4	40.4	φ = 0.074	20.0	50.4	29.6	φ = 0.259
Moderate intensity PA	Decrease	26.8	41.5	31.7	χ^2^ = 4.231	36.5	54.5	9.0	χ^2^ = 23.982
	No change	18.9	47.0	34.1	*p* = 0.376	19.9	65.1	15.1	*p* < 0.001
	Increase	23.1	38.8	38.0	φ = 0.097	24.4	50.4	25.2	φ = 0.229
Walking	Decrease	27.5	35.3	37.3	χ^2^ = 14.639	35.5	50.3	14.2	χ^2^ = 12.133
	No change	17.3	55.6	27.1	*p* = 0.006	19.5	64.7	15.8	*p* = 0.016
	Increase	22.4	38.8	38.8	φ = 0.173	25.4	54.1	20.5	φ = 0.157
Food quantity	Decrease	21.7	36.1	42.2	χ^2^ = 12.947	26.1	56.6	17.2	χ^2^ = 16.045
	No change	18.8	51.0	30.1	*p* = 0.012	18.9	65.0	16.0	*p* = 0.003
	Increase	26.1	35.2	38.6	φ = 0.161	35.7	46.4	17.9	φ = 0.178
Food quality	Worsen	35.0	28.7	36.4	χ^2^ = 35.985	40.4	47.3	12.3	χ^2^ = 31.352
	No change	18.9	52.9	28.2	*p* < 0.001	20.1	65.1	14.8	*p* < 0.001
	Improve	22.1	42.8	35.1	φ = 0.269	21.7	51.2	27.1	φ = 0.249

## Data Availability

All of the data used in the present study can be provided by the corresponding author upon reasonable request.

## Supplementary Materials

The following are available online at https://www.mdpi.com/article/10.3390/ijerph18147292/s1. Table S1: Content of the questionnaire.
