# Supplementary material for: Sleep, Physical Activity, and Diet of Adults during the Second Lockdown of the COVID-19 Pandemic in Greece
Source: Int J Environ Res Public Health. 2021 Jul 8;18(14):7292. doi: 10.3390/ijerph18147292 (PMC8305576; doi:10.3390/ijerph18147292)
Supplement: Supplementary file 1 [file ijerph-18-07292-s001.zip › ijerph-1237594-supplementary.pdf]

International Journal of
Environmental Research and Public Health

MDPI

Supplementary Material

Table S1. Content of the questionnaire.

The items of the questionnaire concerned:<br>(1) sex (women, men),<br>(2) age (years),<br>(3) marital status (unmarried, married),<br>(4) residence (urban, rural),<br>(5) educational level (primary, secondary, tertiary),<br>(6) job (health professionals, with physical presence, full-time distance working, part-time distance working, unemployed, other),<br>(7) compliance with lockdown restrictions (yes, no),<br>(8) infected with COVID-19 for the participant or in their close environment (yes, no),<br>(9) weight (kg),<br>(10) height (m),<br>(11) sleep duration in a typical night of last week (h),<br>(12) quality of sleep in a typical night of last week (bad, average, good),<br>(13) sleep duration in a typical night before lockdown (h),<br>(14) quality of sleep in a typical night of last week compared to a typical night before lockdown (worsen, no change, improve),<br>(15) frequency of high intensity PA during the last seven days (days),<br>(16) duration of high intensity PA during a typical day (min),<br>(17) compared to a normal week, change of high intensity PA in lockdown (decrease, no change, increase),<br>(18) frequency of moderate intensity PA during the last seven days (days),<br>(19) duration of moderate PA during a typical day (min),<br>(20) compared to a normal week, change of moderate PA in lockdown (decrease, no change, increase),<br>21) frequency of walking during the last seven days (days),<br>(22) duration of walking during a typical day (min),<br>(23) compared to a normal week, change of walking in lockdown (decrease, no change, increase),<br>(24) time spent sitting in a typical day during the last seven days (h),<br>(25) change of weight during lockdown (decrease, no change, increase),<br>(26) quantification of change of weight (kg),<br>(27) quantitative change of food consumption in lockdown (less, the same, more),<br>(28) qualitative change of food consumption in lockdown (worsen, the same, improve).
